# Supplementary material for: FluentSigners-50: A signer independent benchmark dataset for sign language processing
Source: PLoS One. 2022 Sep 12;17(9):e0273649. doi: 10.1371/journal.pone.0273649 (PMC9467305; doi:10.1371/journal.pone.0273649)
Supplement: S1 Table — (PDF) [file pone.0273649.s001.pdf]

S1 Table. S1 Table contains the sentences used in this study.

S1 Table. Sentences used in the study (translated to English)

Sentence ID	English translation
S000	Hello
S001	Hi
S002	How are you?
S003	How is your job?
S004	I am doing great
S005	I am all good
S006	I am fine
S007	I am doing terribly
S008	I am very bad
S009	What are you doing?
S010	I am resting
S011	I am working
S012	I am watching TV
S013	How is life? (How is it?)
S014	How are you feeling?
S015	What happened?
S016	I am just tired
S017	Did anything happen?
S018	Nothing, everything is fine
S019	What is new?
S020	I have got news for you
S021	I have news
S022	Nothing is new
S023	Great news
S024	How was your day?
S025	There was a lot to do
S026	Well, I have done a lot today
S027	Where is the toilet?
S028	I need to go to the toilet
S029	How can I help you?
S030	I need help
S031	I will help you
S032	I am looking for a job
S033	I need an interpreter
S034	What to do on the weekend ?
S035	Please, call an ambulance
S036	Please, call the police

Continued on next page

Table 1 – continued from previous page

Sentence ID	English translation
S037	Help, my head and heart hurt and it is hard to breathe
S038	I can not breathe
S039	I need to breathe more
S040	What to do?
S041	Have a nice day
S042	Have a good evening
S043	Good morning
S044	Good afternoon
S045	Good evening
S046	Good night
S047	Take care of yourself
S048	Say hello to your wife
S049	Good luck tomorrow
S050	I wish everything to work out well
S051	Thank you so much
S052	See you soon
S053	We will call each other
S054	Goodbye
S055	What is your name?
S056	My name is
S058	My nickname is
S059	Nice to meet you
S060	Me too
S061	How old are you?
S062	My age is
S064	Where are you from?
S065	Where were you born?
S066	I am from the North of Kazakhstan
S067	I am from the South of Kazakhstan
S068	I am from the West of Kazakhstan
S069	I am from the East of Russia
S070	I live in the city of Almaty
S071	I live in the city of Moscow
S072	I live in the city of St. Petersburg
S073	I live in the city of Tashkent
S074	How long have you lived in Kiev?
S075	Yes, I was born here
S076	No, I live here for now
S077	Yes, I have lived here since my childhood
S078	I have lived here since birth
S079	No, we moved here recently

Continued on next page

Table 1 – continued from previous page

Sentence ID	English translation
S080	When is your birthday?
S081	Today is my birthday
S082	I had a birthday yesterday
S083	It is your birthday tomorrow, congratulations
S084	I am happy for you
S085	Are you married?
S086	I have a spouse
S087	I am married
S088	I am single
S089	Do you have children?
S090	My daughter is beautiful
S091	My wife goes to the store
S092	My husband plays football
S093	Your son is very smart
S094	My son is engaged in wrestling
S095	Your boy and your girl go to swimming classes
S096	Children are playing football
S097	What do you do?
S098	I am a housewife
S099	Where do you work?
S100	I am looking for a job
S101	I work in a garment factory
S102	I work in a cafe
S103	I work as a carpenter in a workshop
S104	My mom works as a hairdresser
S105	My dad is a shoemaker
S106	I will be a student soon
S107	I am in school too
S108	I love animals
S109	I adore dogs too
S110	I have a cat
S111	Do you want coffee?
S112	I love coffee
S113	I do not drink coffee, but I like tea
S114	Let's go outside
S115	Do you want to walk together?
S116	Let's drink coffee
S117	Let's have some tea
S118	I invite you to my home
S119	I need to go home
S120	Are you hungry?

Continued on next page

Table 1 – continued from previous page

Sentence ID	English translation
S121	I want to eat
S122	I want to sleep
S123	Do you like fish?
S124	Do you eat pork?
S125	Yes, I eat everything
S126	No, I do not eat fish
S127	I am always happy to
S128	I will gladly come next time
S129	What is your telephone number?
S130	Do you have an Instagram?
S131	Add me to the VK group
S132	Add me to the group on Facebook
S133	I like you
S134	I do not like you
S135	Let's be friends
S136	My brother and sister are deaf
S137	Do you have deaf relatives?
S138	My mom and dad are deaf
S139	My grandparents are hearing
S140	I have hearing uncle and aunt
S141	I have a deaf child
S142	My brother has lost his hearing
S143	My daughter was born deaf
S144	I want to practice my sign language
S145	Super, I will teach
S146	I will help you with pleasure
S147	I am sorry I am just starting to learn sign language
S148	It is okay, there is time
S149	I know sign language a little bit
S150	Unfortunately, I do not understand
S151	You are gesticulating too quickly
S152	Please take your time
S153	Sorry, I am not following
S154	Can you please slow down
S155	Can you please repeat?
S156	What is a sign for ... (point to object)
S157	Sorry I do not know
S158	I am a little worried
S159	Are you deaf?
S160	Excuse me
S161	Forgive me

Continued on next page

Table 1 – continued from previous page

Sentence ID	English translation
S162	I am sorry
S163	I am so sorry
S164	Everything is OK
S165	Do not forget to continue practicing, study well
S166	What a wonderful day
S167	Today is so hot
S168	What is the weather right now?
S169	Is it snowing outside?
S170	Good weather
S171	It is very cold outside
S172	I love when it rains
S173	I do not like the heat
S174	I like the wind
S175	There is a very strong wind outside
