# Supplementary material for: FluentSigners-50: A signer independent benchmark dataset for sign language processing
Source: PLoS One. 2022 Sep 12;17(9):e0273649. doi: 10.1371/journal.pone.0273649 (PMC9467305; doi:10.1371/journal.pone.0273649)
Supplement: Attachment — Submitted filename: Response to Reviewers_ krsl dataset PLOS ONE .pdf [file pone.0273649.s002.pdf]

Anara Sandygulova

Nazarbayev University
Kabanbay Batyr Avenue, 53
anara.sandygulova@nu.edu.kz
+7 777 957 99 00

Dear editor Aaron Jon Newman and the reviewer,

We are grateful for your time and reviews. Below we address the points raised by the reviewer and the editor and describe the changes we implemented. All the implemented changes are highlighted with blue text color in the marked up copy of the manuscript.

Concerning Figure 1, we obtained permission from the participants for their photo to appear in an open access publication. All signed consent forms are archived and available upon request.

We have also checked that the manuscript meets the PLOS style requirements.

Acknowledgement- we removed funding information from this section and changed acknowledgement to “We would like to thank the participants who kindly agreed to contribute to the dataset and have their recordings shared publicly.“ They are available upon request.

Data availability - we apologize for not sharing the data at the review process. We now shared our dataset at the following DOI: 10.5281/zenodo.6043739. Due to the size constraint, we only share one repetition by each contributor. A full version with five repetitions per person are located at the link: https://krslproject.github.io/fluentsigners-50/

We address each comment in details below.

> **Reviewer #1:** The paper presents a new dataset of Kazakh-Russian Sign Language from 50 native signers. The dataset contains native signers, different ages, different devices. Hence, it is challenging.
>
> “However, the dataset is not available. The link provided is empty. I would like to inspect the dataset, the annotations, etc. The annotation protocol is not provided.”

We apologize for not uploading the dataset on time. We made the dataset available at this link: https://krslproject.github.io/fluentsigners-50/. Its DOI is 10.5281/zenodo.6043739.

> The dataset has 3 splits: Signer independence, age, and unseen sentences. Split 1 has 2 validation subject, 5 test subject and the rest is training. Is it just one fold or several folds? If so, how many. 1 fold is not enough because the selected 5 subjects may be too easy or too hard. Ideally, one must have, say, 10 folds, random 5 subjects in test each time. Then, one can report mean performance figures and variance.

Thank you for pointing this out. We followed the same methodology as the RWTH-PHOENIX-Weather-2014T [1] dataset as it is considered as the sign language recognition and translation benchmark. This dataset has one split into training, validation, and test sets for people to have comparable results on the same videos. It was also the case for the Chinese Sign Language (CSL) datasets [2]. This seems to be a common practice for sign language recognition tasks with one shared test set for more comparable results.

We have also included the proposed splits (train, val and test sets) at the link shared above. We included this explanation in the paper under “Suggested Splits”.

Nevertheless, we decided to address your suggestion by conducting a 5-fold cross-validation on Split 1 and Signer Independent Split 3. Unfortunately, we could not do it on Split 2 as they are not suitable for creating five-folds due to not having enough data points to satisfy splits’ definitions. Tables 4 and 6 are updated with the newly obtained results.

References:

1. Camgoz, N. C., Hadfield, S., Koller, O., Ney, H., & Bowden, R. (2018). Neural sign language translation. In Proceedings of the IEEE conference on computer vision and pattern recognition (pp. 7784-7793).
2. Huang, J., Zhou, W., Zhang, Q., Li, H., & Li, W. (2018, April). Video-based sign language recognition without temporal segmentation. In Proceedings of the AAAI Conference on Artificial Intelligence (Vol. 32, No. 1).

> The paper is nicely written. However, figures could be better quality with informative captions. It would be good to have successful and failing examples in split 3 as a figure.

Thank you for your suggestion. We modified the captions of all figures to be more informative per your suggestion. We also included Table 5 with successful and failing examples in Split 3.

> The term "signed speech" should be replaced with "sign language". You have used it once in page 1 and then at the end of page 5. Signed speech is signing each word in the order of spoken language, simultaneously with spoken language. This dataset is sign language, not signed speech or signed language.

Thank you for your comment. We updated the paper to avoid using signed speech.

> The paper uses the RWTH-PHOENIX-Weather-2014T dataset as a sign language recognition and translation benchmark.
> Stochastic CSLR is used as a benchmark for continuous SLR. 26.1 WER is obtained on Phoenix.
> TSPNet is used as a segmentation benchmark for sign language translation using multi scale attention. 13.41 Bleu4 score is obtained.
> The results for the RWTH-PHOENIX-Weather-2014T dataset are the same numerical values from the respective papers. Did you print the numbers from the authors' papers directly or did you train your methods on these methods and confirm these numbers?

Thank you for your question. We printed the numbers from the authors’ papers directly. We included this detail in the paper.

> Camgoz et al. (Sign Language Transformers: Joint End-to-end Sign Language Recognition and Translation) obtains 24.5 WER and 21.8 BLEU4 score on the same 2014T dataset in 2020. You already have the reference in your literature section, but omitted the results.

Thank you for your comment. We included the results in the literature section for Camgoz et al. (2020) and other related works similar to what we did for the Sign Language Recognition section. Although Camgoz et al. obtained a higher BLEU4 score on the 2014T dataset, we selected TSPNet as the state-of-the-art method for video-to-text task. Camgoz et al. uses as an intermediate step of segmenting

video into glosses and then translating them into text, while TSPNet proposes novel sign video segment representation alleviating the need for accurate video segmentation. We believe that this approach has a more applicable potential as not every dataset has gloss-based annotations. And despite its current lower performances compared to Camgoz et al., TSPNet can accommodate more datasets that have only subtitles to videos. We included this justification in the paper.

Why does split 3 obtain better BLEU1-3 scores than split 1? WER scores from the first method do not correlate with these results. Is signer dependence the cause of this? If so would removing sentences from the same user make split 3 more realistic?

Thank you for your suggestion. We decided to follow it and replace our current Split 3 with the proposed Split 3 to be signer independent. This makes Split 3 even more challenging, we included its 5-fold results for both CSLR and SLT. For each fold, our testing set contains 10 unseen signers doing unseen sentences.

Some minor errors to be corrected:
-recognizing a real-life signed speech --> recognizing real-life sign language
-were born and grown up --> were born and grew up
- 173 translations --> 173 sentences
- too many uses of "etc."

Thank you for pointing out these errors. We corrected them in the paper.

Editor comments:
- The motivation for this work is not clear. Please state more clearly the goals of: (a) automated sign language (SL) recognition; (b) automated SL translation; (c) developing corpora to train these. With regard to the last point, is the goal of developing a KRSL database to support the field of SLP generally, or more narrowly SLP related to KRSL? What kind of generalizability is desirable?

Thank you for your comment. We updated the text in Introduction to answer these questions.

- Related to the above question, please provide a better rationale for a corpus that includes a wide range of cameras, backgrounds, and other recording details. If the purpose of a corpus is to train SLR/SLT systems to achieve high accuracy, then wouldn't more uniform recording conditions be preferable?

Thank you for your question. Obtaining higher accuracy on uniform recording conditions will make the computer vision problem easier, and thus less robust when deployed in real-world applications (e.g. smartphone application). Having a richer recording variability and environmental settings within the dataset will result in a better performance of a trained model when it is tested with new unseen data.

- There are a few questions around "native signers". It is true that, like all natural human languages, native signers should be the standard in determining what "correct" SL is, and that non-native signers are likely to produce more variable and nonstandard signing. However, it is not clear that restricting training coprora for automated SLR and SLT tasks to native signers is appropriate. This would seem to limit their generalizability, and indeed due to the variability of when people become deaf, and when, relative to becoming deaf, they learn a SL, and the historic patterns of suppression of SLs in educational systems (e.g., the residential school systems in the US and Canada), there are many more non-native than native signers in most SL communities. As a result, there is greater variability amongst signers in how signs are produced.

As such, one would predict that a system trained only on native signing would generalize quite badly to much natural SL input. The question/request here is, please provide a discussion of why restricting a corpus like this to native signers is appropriate, and what the limitations are.

Thank you for your question. The main goal of this dataset was to involve Deaf community members to its data collection to obtain a signer variability as well as natural sign language input. However, as rightly pointed out by you, the models should be robust to recognition of various signers present within the Deaf community (native or not native). It was misleading to the reviewers to name the dataset as NativeSigners-50 as we were not accurate in the definition of what it means to be native. What we really wanted to stress is their level of sign language usage (i.e. daily, fluency, etc.) to make the recognition models applicable to Deaf community and in contrast to datasets that record convenient contributors who are university students and have no knowledge of sign language.

In addition, another point is that not all of our contributors were native to KRSL. To acquire their status, we distributed a survey to clarify their KRSL status. The question was borrowed from Allen (2015) and was the following:

To the best of your memory, or from what your parents have told you, which of the following best describes your use of sign language in your home during your early childhood?

- We only signed and used no spoken language.
- We mostly signed, but we used some spoken language as well.
- We signed and spoke in roughly equal amounts.
- We mostly spoke, but used some sign language too.
- We only spoke and used no sign language.
- We rarely spoke or signed, but relied on gestures to communicate

Do you use sign language on a daily basis?
- Yes
- No

Additionally, we explicitly asked when they learned sign language:
- from birth
- In kindergarten
- In school
- In adulthood

You can find the results of the survey at the link:
https://docs.google.com/forms/d/1xBPdacqdP8LQVzHYzbdx1CtcQABWP0esJ3r9U9A8RF0/edit?usp=sharing

As can be seen there, it appears that 16 contributors are not native as they learned SL in kindergarten (N = 4), school (N=9) and adulthood (N=3). Therefore, we propose to name the dataset as FluentSigners-50.

References:

1. Allen, T.E. (2015). The Deaf Community as a “Special Linguistic Demographic”. In Research Methods in Sign Language Studies (eds E. Orfanidou, B. Woll and G. Morgan). https://doi.org/10.1002/9781118346013.ch2

Challenges

- p 3 contains the sentence, "Many datasets record professional interpreters who are often not native signers (i.e., CODA)". Being a CODA is not an acceptable definition of "native signer". Firstly, children of deaf adults may not be native signers because their parents don't sign. Secondly, one could be a native signer by virtue of being born to hearing parents who do sign (e.g., the child of two CODAs). Please revise this sentence, and provide an accurate definition of "native signer".

Thank you for your clarification of the “native signer” definition. We revised this sentence on p.3: Many datasets record professional interpreters performing interpreting tasks. The nature of the task might lead them to use calque or loan translation, or the signed version of the spoken language instead of the natural sign language. Furthermore, many interpreters acquire sign language as adults, which also has important consequences to their sign language production.

- The text of the manuscript is not clear on a very important point - the "native" status of the signers who contributed. Again, being born to a deaf parent is not a guarantee that the child is a native signer, since the parent may not sign. Thus on p. 5 the description, "They are native to KRSL since they were born and grown up in families with at least one deaf parent." is not an acceptable definition of a native signer. Please provide detail on how it was determined/confirmed that each contributor was a "native" signer.

Thank you for clarifying this for us. The sentence on p.5 is revised to avoid using the wrong definition of “native” status. The updated version is the following: “They were born and grown up in families with at least one deaf parent (i.e. hearing CODA).”

Regarding the “native” status of our contributors, we distributed a survey to clarify their status. As reported previously, our contributors were indeed not native. Therefore, we decided to name the dataset as FluentSigners-50.

- Related to the above, the sentences, "According to this distinction, NativeSigners-50 has 30 CODA contributors (including nine hearing signers) and 20 who are not CODA (16 deaf, one hard of hearing, and three hearing SODA). Nevertheless, we decided to name our dataset “NativeSigners-50” because all of our contributors use sign language daily, and it is their primary language of communication." are problematic. If you believe that 20/30 contributors are not native signers, then it is misleading and frankly incorrect to title your database "NativeSigners-50". It would be more appropriate to remove "native" from the title or use a numeral that accurately reflects the number of native signers who contributed.

We agree that your suggestion and we decided to rename the dataset as FluentSigners-50.

- It seems problematic that you apparently created signs in a written language, then translated them into KRSL. SLs have their own grammars and so translations from spoken languages can result in constructions that would not be produced by native signers. Please clarify how this was addressed.

It was a tradeoff that we had to make in order for the dataset to have the same KRSL sentences. Although this approach does not result in constructions that would typically be produced by native signers, we had to rely on the interpreters’ opinions and translations. In addition, the contributors were allowed to make changes to the original interpretation that often included changing the order of signs, omitting or replacing signs, adding extra signs as well as varying the way the signs were performed. As a result of this process, the dataset contains a high degree of linguistic variability.

- Text makes statements that are unsupported by references. For example, p 3 states, "Many datasets record professional interpreters who are often not native signers (i.e., CODA)." But fails to provide citations to clarify what "many" is or what datasets are in question here.

Thank you for pointing this out. We added a citation to a new version of this sentence. “Many datasets record professional interpreters whose act of interpretation might change the execution (e.g., use of a calque or loan translation, i.e., a literal word-for-word translation)”. (Bragg et al., p. 21)

Reference:

1. Bragg, D., Koller, O., Bellard, M., Berke, L., Boudreault, P., Braffort, A., ... & Ringel Morris, M. (2019, October). Sign language recognition, generation, and translation: An interdisciplinary perspective. In *The 21st international ACM SIGACCESS conference on computers and accessibility* (pp. 16-31).

- the description of approaches to SLR on p. 4 is too short, and as a result, confusing. It conflates feature extraction methods with training approaches, and reads more like a list of papers and what algorithm each used, than an explanation of the approaches, their advantages, and their limitations. This section should be revised to be more clear and detailed.

Thank you for your comment. We have added a more detailed explanation for approaches to the SLR section.

- The scope and significance of the sentence on p. 4, "All the evaluations were performed on the RWTH-PHOENIX-Weather 2014 [5] and RWTH-PHOENIX-Weather 2014T [15] datasets.", is unclear. What do you mean by "all the evaluations"? Do you mean all of the papers that you cited in this section, or only the Koller et al reference. And, what is the point of stating this? Are you implying that these data sets are considered a benchmark in the field?

The point of this sentence is to acknowledge that RWTH-PHOENIX-Weather 2014 [5] and RWTH-PHOENIX-Weather 2014T [15] datasets have indeed become a benchmark dataset. The two references below also acknowledge that by referring to it as a community benchmark dataset. We added these references to support our statement.

Reference:

1. Bragg, D., Koller, O., Bellard, M., Berke, L., Boudreault, P., Braffort, A., ... & Ringel Morris, M. (2019, October). Sign language recognition, generation, and translation: An interdisciplinary perspective. In *The 21st international ACM SIGACCESS conference on computers and accessibility* (pp. 16-31).
2. Koller, O. (2020). Quantitative survey of the state of the art in sign language recognition. *arXiv preprint arXiv:2008.09918*.

- Related to the previous point, please provide a rationale for using the RWTH-PHOENIX-Weather 2014 data set as a benchmark

In order to provide a rationale, we are going to quote Koller (2020), “RWTH-PHOENIX-Weather with a vocabulary of 1080 signs represents the only resource for large vocabulary continuous sign language world wide”.

Reference:

1. Koller, O. (2020). Quantitative survey of the state of the art in sign language recognition. *arXiv preprint arXiv:2008.09918*.

- Another limitation of the SLR and SLT sections on pp. 4-5 is that they do not provide the reader with any information on what benchmark levels of performance are, or what is considered "state of the art" in this field. As a result, when you describe your own methods, it is completely unclear why you chose the Stochastic CLSR and TSPNet approaches - indeed, you didn't even introduce these approaches in the "related work" section so the reader has no context as to what these are, why they were chosen, or how they compare to other approaches.

Thank you for your comment. We followed your suggestion and added missing methods to the related work section.

- Typically in ML, one defines the proportion of items assigned to the training, validation, and test sets a priori as percentages of the total items (e.g., 80-10-10). In the present case, (Splits 1 and 2) it does appear that your test set comprises 10% of your samples (although this is never expressed as a percentage); however your validation comprises only 4% of the data. Please provide a rationale for this low number.

Thank you for your comment. We followed your suggestion and changed the percentages to 80-10-10 in Split 1. Similar to what we previously discussed, Split 2 and 3 can not have such proportions. However, given the scale of the dataset, we consider our validation to be large enough for hyperparameter optimization and choosing the best model. Although we have included the validation and testing sets for each split, researchers are free to split the dataset into train and validation sets as long as the model results are reported on the fixed unseen testing set.

- Please clarify how the RWTH-PHOENIX-Weather 2014[T] data set was used. Did you divide it into training, test, and validation sets? If so, how and in what proportions?

The results from the original paper by Koller et al. were reported in our tables. The dataset has one fixed split proposed by the original authors. We downloaded and used their training, validation, and test sets.

- I agree with R1's concerns regarding using a single split of each type. This raises significant concerns about generalizability, because your results in the present case are entirely dependent on the choice of what items were assigned to which set. By using a number of different folds you would increase generalizability, as well as the reader's confidence that your results were robust.

Similar to our response to R1’s concern, we would like to acknowledge that you are right, and using a number of different folds increases the generalizability and robustness of the results. However, such n-fold cross-validation is typically done for small datasets where the computation can be done quickly. In our case, the dataset is relatively large and each sample is a video, which is computationally intensive even for powerful GPUs (in our case Tesla V100, 32GB). Considering that similar datasets such as RWTH-PHOENIX-Weather 2014[T] and Chinese Sign Language (CSL) datasets have one fixed testing set, it seems reasonable to have a fixed split.

As stated earlier, we decided to conduct a 5-fold cross-validation on Split 1 and Signer Independent Split 3. The results are updated in Tables 4 and 6.

- Please provide more details on your methods. For instance, it is insufficient to say that "Before passing through the model, each raw image is preprocessed." What preprocessing steps were performed?
- please provide a citation for the "end2end architecture"

Thank you for pointing this out. This was updated in the paper.

- please ensure that all acronyms and abbreviations are defined, and do so the first time they are used (e.g., WER, CTC)

We have checked all acronyms and abbreviations and have added missing definitions.

- I am unclear on the reason for discussing the fact that "n-gram BLEU scores rapidly increase as n decreases from 4 to 1". This seems intuitive, since any training set will naturally include fewer repetitions of any 4-gram than 3-gram, etc. - and as noted, this is a property of the results on the RWTH-PHOENIX-Weather 2014T data set as well. Thus, your point seems irrelevant to your paper or your dataset (although it doesn't drop as dramatically in Splits 1 or 2). Perhaps your point has to do with the ability to generalize to signs/phrases not seen in the training set, however if so this needs to be discussed more clearly and explicitly.

To address R1's comment, we used Split 3 with signer independence which did not result in such performance.

- I also disagree with the statement that, "the results of TSPNet on RWTH-PHOENIX-Weather 2014T demonstrate similar behavior of rapid increase (13.41 increasing to 16.88 to 23.12 to 36.10) but with a much better BLEU-4 score (13.41 vs. 3.08)." - because you are selectively reporting only the NativeSigners-50 result for the poorest-performing split; the performance on Split 1 is actually higher than for RWTH-PHOENIX-Weather 2014T.

Thank you for pointing this out. We removed this sentence.

- Please provide some discussion of how the SLR and SLT results compare between your data set and the RWTH-PHOENIX-Weather 2014[T] one. You present comparisons of these in tables, but the significance of these is never discussed. Given that you refer to "potential limitations" of RWTH-PHOENIX-Weather 2014[T], how do you reconcile the fact that SLR/SLT performance was in general very similar between the two data sets? What would the benefit be to researchers of employing your suggestion that they report results of training applied to both your and this other data set?

These datasets serve different purposes as our dataset contributes with its signer independence while RWTH-PHOENIX-Weather 2014[T] has limitations in the number of signers and variability in the recording conditions. Additionally, our dataset proposes more challenging splits (2 and 3).

- please correct the text on lines 126-127 of p. 4, which appears to be a mistaken pasting of text, or some sort of formatted article generation error.

Thank you for pointing this out. We removed this error.
