# Supplementary material for: FluentSigners-50: A signer independent benchmark dataset for sign language processing
Source: PLoS One. 2022 Sep 12;17(9):e0273649. doi: 10.1371/journal.pone.0273649 (PMC9467305; doi:10.1371/journal.pone.0273649)
Supplement: Attachment — Submitted filename: Response to Reviewers 2_ krsl dataset PLOS ONE .pdf [file pone.0273649.s003.pdf]

Anara Sandygulova

Nazarbayev University
Kabanbay Batyr Avenue, 53
anara.sandygulova@nu.edu.kz
+7 777 957 99 00

NAZARBAYEV UNIVERSITY
SCHOOL OF ENGINEERING AND DIGITAL SCIENCES

Dear editor and the reviewers,

We are grateful for your time and reviews. Below we address the points raised by the reviewers and describe the changes we implemented. All the implemented changes are highlighted with blue text color in the marked up copy of the manuscript.

We address each comment in details below.

Reviewer #2:

1. in Paper [1] did they mentioned 300 distinct sign languages?

Thank you for pointing this out. Yes, they analyzed 300 works on sign language recognition, not 300 sign languages. We corrected this text in the paper.

2. Comparison with RWTH-PHOENIX-Weather database is not convincing?

According to [1], RWTH-PHOENIX-Weather dataset is referred to as “the only resource for large vocabulary continuous sign language recognition benchmarking world wide”, although it has its own limitations. As the goal of our work is to provide an alternative dataset that could be used for sign language recognition tasks, a comparison to RWTH-PHOENIX-Weather is required. This way, the researchers can test their models’ performance on unseen signers and new sign languages.

3. Paper formatting needs attention.

Thank you. We tried to address formatting issues.

Reviewer #3:

> The paper presents a dataset of Russian sign language. This is a valuable resource as we need to recognize, document, and study different sign languages around the world. The paper needs major revisions before it’s ready for publication.
>
> It needs clear arguments and explanations for how it can be used. What are the kinds of questions that people can answer with this dataset? Is this going to be good for linguistics and cognitive science studies for coreference resolution for example? Is the quality of the facial gesture good enough for sign simultaneously modeling? Is it only good for certain computer vision modeling tasks?

The main purpose of this dataset is to be used as a benchmark for sign language recognition/translation architectures. It can help researchers find out if their proposed models perform well and can generalize on unseen signers and different sign languages. Additionally, the dataset can be of interest to sign language linguistics as it has real-life, linguistic and inter-signer variability. Sentence types include statements, polar questions, wh-questions, and requests. This can allow linguists to analyze the data regarding its sentence type or non-manual features. Although the dataset has its own shortcomings, we believe that it is necessary to provide alternative datasets for ML

purposes to be used by the community working on sign language recognition and translation tasks. We added these details to the paper.

> The paper needs lots of screenshots, figures, and examples so the reader can learn about the context, use-case, environment of the data collection, etc. In its current form, it’s really hard for readers to understand the context and details of the dataset.

Thank you for your suggestion. We have updated the figures and added more visual examples from the dataset.

> The anonymity issue is not discussed. If the dataset is going to be bale publicly, how are anatomizing it? What kinds of consent forms do researchers need to sign in order for them to be ale to work with the facial gestures?

The contributors agreed and signed consent forms for the dataset to be released publicly. It is freely available to be used for research and development purposes.

> How many different Russian sign language dialects exist? How diverse is your dataset? What are the limitations?

Russian Sign Language (RSL) is the signed language used in Russian Federation; it has dialectal variation even though it is understudied at the moment (see e.g. https://www.frontiersin.org/articles/10.3389/fpsyg.2021.740734/full). The signed language used in Kazakhstan, which is under investigation in the current study, is, as discussed, very close to RSL lexically and most likely grammatically, which is why we use the name KRSL for it. Some other ex-Soviet countries also have sign languages that were heavily influenced by or closely related to RSL, but this is not relevant for the analysis of KRSL, so we do not discuss it. Finally, the issue of dialectal variation of KRSL in Kazakhstan has not been studied at all. As mentioned in the paper, the signers in the dataset come from different regions of Kazakhstan, so some regional variation is possibly represented in the dataset. However, it was not created for the purposes of studying dialectal variation. We added this explanation to the paper. Thank you for your question.
